# Genomics and Physiology of Chlorophyll Fluorescence Parameters in *Hordeum vulgare* L. under Drought and Salt Stresses

**DOI:** 10.3390/plants12193515

**Published:** 2023-10-09

**Authors:** Somayyeh Makhtoum, Hossein Sabouri, Abdollatif Gholizadeh, Leila Ahangar, Mahnaz Katouzi, Andrea Mastinu

**Affiliations:** 1 Department of Plant Production, Faculty of Agriculture Science and Natural Resources, Gonbad Kavous University, P.O. Box 163, Gonbad 49717-99151, Iran; somayyeh.makhtoum@yahoo.com (S.M.); lgholizadeh@gmail.com (A.G.); l.ahangar63@gmail.com (L.A.); 2 Department of Plant Breeding and Genetic Resource, Agroscope, 1260 Nyon, Switzerland; mahnaz.katouzi@agroscope.admin.ch; 3 Department of Molecular and Translational Medicine, Division of Pharmacology, University of Brescia, Viale Europa 11, 25123 Brescia, Italy

**Keywords:** barley, chlorophyll fluorescence, salinity, drought, molecular markers, quantitative trait loci

## Abstract

To map the genomic regions and control chlorophyll fluorescence attributes under normal, salinity-, and drought-stress conditions in barley (*Hordeum vulgare* L.) at the seedling stage, an experiment was conducted in 2019–2020 using 106 F8 lines resulting from the cross between Badia × Kavir. Initially, the different chlorophyll fluorescence parameters were evaluated. Under drought stress, the highest decrease was related to REo/CSm (59.56%), and the highest increase was related to dV/dto (77.17%). Also, under salinity stress, the highest decrease was related to Fv/Fo (59.56%), and the highest increase was related to DIo/RC (77.17%). Linkage maps were prepared using 152 SSR polymorphic markers, 72 ISSR alleles, 7 IRAP alleles, 29 CAAT alleles, 27 Scot alleles, and 15 iPBS alleles. The obtained map accounted for 999.2 centi-Morgans (cM) of the barley genome length (92% of the whole barley genome). The results indicated the importance of chromosomes 3, 2, and 7 in controlling ABS/CSm, Area, ETo/CSm, Fm, Fv, and ETo/RC under drought stress. qEToRCD-7, as a major QTL, controlled 18.3% of ETo/RC phenotypic variation under drought stress. Under salinity stress, the regions of chromosomes 2 and 7 (102 cM and 126 cM) controlled the parameters ABS/CSo, Fm, Fo, Fv, TRo/SCo, Area, ETo/CSm, and ETo/CSo. The results showed that chlorophyll fluorescence is an important parameter in the study of drought and salinity effects on barley. This is the first report of the investigation of changes in the genetic structure of quantitative genes controlling the fluorescence parameters associated with barley response to drought and salinity stresses in the Iranian barley RILs population. According to the obtained results, it is possible to use HVPLASC1B and EBmac0713 in normal conditions, ISSR21-2 and ISSR30-4 in drought conditions, and Bmac0047, Scot5-B, CAAT6-C, and ISSR30iPBS2076-4 in saline stress conditions to select genotypes with higher photosynthetic capacity in marker-assisted selection programs.

## 1. Introduction

Barley *(Hordeum vulgare* L.), owing to its wide ecological range, area under cultivation, and distinct ecological compatibility compared to other crops, can be grown under a variety of climatic conditions [1]. This crop is believed to be one of the most important species that is resistant to environmental stress. However, the growth and yield of barley in many parts of the world are strongly affected by these stressors, causing severe economic losses for producers [2]. Biotic and abiotic stresses are important factors that affect plant production and food security worldwide [3]. Drought causes dehydration of plant cells, stomatal closure, and low stomatal conduction by inhibiting metabolism and reducing photosynthesis, leading to permanent wilting of the plant [4]. Also, a reduction in plant morphology (reduced leaf size and stem length, leaf length/width, and vegetative growth) and physiological traits (reduction in photosynthesis parameters and leaf water potential) have been discovered [5,6]. The effects of drought and excessive light disrupt the photochemical functions of photosystem II, inhibiting electron transfer [5]. Salinity is one of the most important factors that reduce crop yield, particularly in arid and semi-arid regions of the world [7]. NaCl salinity reduces carbohydrates, which are essential for cell growth and the main stages of photosynthesis [8].

Among the various traits effective in plant tolerance to non-biotic stresses, chlorophyll fluorescence has received much attention today. Chlorophyll fluorescence is an important factor in leaf photosynthesis. Chlorophyll fluorescence can effectively measure the environmental stresses and help to determine drought resistance in crop and fruit crops. [9]. Today, chlorophyll fluorescence, as a measurement criterion for measuring the effect of environmental stresses, has been suggested to include water stress in crops and to determine their drought resistance [10]. Chlorophyll fluorescence is commonly used to evaluate the response of plant species to stresses such as frost, salinity, and drought [11]. Although measuring the chlorophyll fluorescence parameter is a simple process, it requires special care. In addition, it does not degrade the plant tissues. In this method, the Fv/Fm ratio represents the maximum quantum efficiency of photosystem ІІ and a measure of the optical system function. The value of this parameter decreases in most plant species when plants are exposed to stress [12,13]. Environmental stress reduces this ratio by affecting photosystem II. Barley salinity-tolerant cultivars had a higher Fv/Fm ratio than susceptible cultivars. In other words, the efficiency of optical system II was higher in the resistant cultivar [14]. Higher chlorophyll fluorescence under drought stress has been reported in many crops, including strawberries, lettuce, and olives [15,16,17].

Plant growth depends on the amount of net photosynthesis, and environmental stress affects photosynthesis depending on its intensity. Low ion concentrations also increase the intensity of photosynthesis [18]. ROS are produced when the plant is exposed to stress conditions. The plant produces antioxidants, flavonoids, and secondary metabolites that play the role of protecting the plant from detoxifying ROS and protecting the plant from abnormal conditions (i.e., stress) and protein and amino acid stabilization [19].

Drought stress reduces chlorophyll, plant growth, total soluble proteins, yield per plant, oil percentage, and organic contents of palmitic and linoleic acids in sunflowers [20], and aging is a complex process that involves the development and reduction in leaf chlorophyll in the early stages. The progression of leaf aging causes the redistribution of nutrients to the younger leaves [21]. Drought reduces chlorophyll content and fluorescent components (Fv/Fm) but increases electrolyte leakage and H_2_O_2_ in plants [22]. Kaya et al. clearly showed that combined stress (water stress and phosphorus deficiency) significantly reduced maize yield and fluorescent components.

Chlorophyll a and chlorophyll b content decreased significantly under water stress. Ascorbic acid significantly improves the chlorophyll content (a, b) under water stress [23]. Drought stress also reduces the synthesis of green pigments (chlorophyll content), thereby reducing the rate of photosynthesis [24]. Chlorophyll fluorescence parameters (Table 1) comprise three parts: light absorption (ABS), excited energy trapping (TR), and excited energy transfer to the electron transfer chain (ET) through the reaction center (RC). Due to the changes in the behavior of photosystem II under environmental stress, the physiological parameters of this photosystem could be analyzed under different environmental stress conditions using the O-J-I-P (chlorophyll fluorescence induction) test [25].

Identifying the genetic structure of chlorophyll fluorescence parameters can provide suitable solutions for the genetic modification of barley.

An experiment was conducted to determine the genomics controlling of physiological traits of double haploids populations from the crossing cultivars Steptoe and Morex barley under normal conditions and salinity stress [27]. The results obtained through the composite analysis of variance showed significant differences between the lines for all attributes. The maximum correlation was observed between Fo and Fv/Fm. For the studied traits, 43 loci with QTLs 13, 16, and 14 were detected for non-stress conditions, salinity stress, and their mean, respectively. Phenotypic variances explained by these QTLs ranged from 9.06 to 30.28% for Fo (qFo1m) and Fv/Fm (qFvFm2.1m); the mean of control and non-stress conditions indicated variable salinity stress.

Tavakkoli et al. evaluated the effect of salinity on the photosynthetic efficiency of barley cultivars (Dasht, Sahand, Lisvi, and Sahra) under hydroponic conditions and different concentrations of sodium chloride (NaCl) (50 and 100 mM) [28]. Various chlorophyll-a fluorescence parameters, including Fo, Fm, Fv/Fm, AREA, PI, DI/RC, RC/CS, TR/ABS, and DI/ABS, and the number of photosynthetic pigments were measured. All pigments decreased following the salinity treatment. With an increase in stress, chlorophyll-a fluorescence increased in the cultivars studied and significantly decreased the efficiency of the photosynthetic apparatus.

Salinity stress (NaCl stress) decreases Fv/Fm, IIPSII, ϕexc, and qP but increases NPQ compared to control plants. External supplementation with KN restores chlorophyll fluorescence in plants under salinity stress by increasing Fv/Fm, IIPSII, ϕexc, and qP [29].

Ahanger and Agarwal showed a significant effect of stress cell repeated membrane leakage significantly affects chlorophyll biosynthesis in *Triticum aestivum* [30,31].

Photosynthetic efficiency decreases with decreasing salinity in chlorophyll fluorescence parameters, and KN improves plant yield by increasing Fv/Fm, IIPSII, ϕexc, and qP and decreasing NPQ. Stress is believed to damage plant antenna pigments, thus limiting electron transfer from PSII to PSI [32].

Yin et al., using 184 recombinant inbred lines (RILs) detected the fluorescence parameters MF (Fv/Fm, Fv’/Fm’, UPSII and qP) and PN (photosynthetic rate) [33]. Four important genomic regions were identified: LGA2 (19.81 cM) for JIP test parameters; LGC1 (94.31 cM) for PN and MF parameters; LGM (97.61–100.51 cM) for JIP and MF parameters; and LGO (30.61–49.91 cM) for PN, JIP, and MF parameters. These results showed that chlorophyll-dependent parameters, particularly UPSII and the photochemical quenching coefficient qP, could play an important role in regulating PN.

Guo et al. detected QTLs controlling chlorophyll content and fluorescence using 194 recombinant inbred lines (RILs) from crossing Arta × *Hordeum spontaneum* 41-1 [34]. Nine and five genomic regions were identified under the water stress and drought conditions, respectively. A QTL for Fv/Fm under drought conditions was detected on chromosome H2 at 116 cM on linkage maps. This QTL alone accounted for over 15% of the phenotypic variation in the maximum PSII quantum performance and was also associated with the expression of four other traits. The results of this study indicate the existence of two important positions during the flowering and post-flowering stages to withstand drought stress in barley under drought stress.

Considering the importance of abiotic stresses, chlorophyll fluorescence as an indicator of energy excitation in leaf photosynthetic structures, and a non-destructive diagnostic system for determining plant resistance to environmental stresses, this study was conducted to investigate the variations in the genomic control regions of chlorophyll fluorescence parameters under normal conditions, drought stress, and salinity. This is the first investigation of the genetic structure of chlorophyll fluorescence parameters of Iranian barley genotypes. This is the first report in which the drought and salinity stresses are investigated simultaneously in an F8 Iranian barley population (Badia and Kavir crosses).

## 2. Results and Discussion

### 2.1. Correlation between Traits and Grouping between Lines

Fm, Fv, Fv/Fm, Fv/Fo, Vi, Sm, TRo/RC, ETo/RC, TRo/CSo, ETo/CSo, ABS/CSm, TRo/CSm, ETo/CSm, and Reo/CSm showed a decrease under drought and salinity stresses compared with normal conditions, whereas the area, dVdto, Fo, ABS/RC, DIo/RC, ABS/CSo, DIo/CSo, DIo/CSm, and Vj increased (Table 2).

Under drought-stress conditions, the highest decrease was related to REo/CSm (59.56%), and the highest increase was related to dVdto (77.17%). After dVdto were DIoCSo and DIo/RC, respectively, followed by REo/CSm, TRo/CSm, Fv, Fm, and ABS/CSm. Also, under salinity stress, the highest decrease was related to Fv/Fo (59.56%), and the highest increase was related to DIo/RC (77.17%). After DIo/RC was ABS/RC, and after Fv/Fo were Fv, Fm, and TRo/CSm.

Plant chlorophyll content and maximum photochemical efficiency (Fv/Fm) are both key characteristics that indicate photosynthetic activity, but under stress conditions are affected deleteriously [22,23,29,30,31,35,36].

In plants, chlorophyll fluorescence parameters are often considered an important tool to determine the effect of environmental stresses on the photosynthetic machinery [37,38]. In general, abiotic stresses, including salinity stress, reduce Fv/Fm, ΦPSII, Φexc, and qp in different plants [39]. The decrease in Fv/Fm under salinity stress may be due to a reduction in the energy transfer efficiency from antennas to reaction centers [37,38]. Decreasing Fv/Fm indicates vital destruction of photosystem II, which may reduce the photosynthetic rate, which in turn can reduce plant growth and yield [12]. A decreased Fv/Fm ratio is believed to disrupt photosystem II activity, thereby causing non-photochemical quenching owing to reduced light energy utilization [39].

Raja et al. (2020) showed that drought and heat stress decreased the efficiency of PSII (Fv/Fm), quantum yield (ΦPSII), and photochemical efficiency (qp) and increased the level of non-photochemical quenching [40]. Compared to the control, all parameters related to photosynthesis showed a sharp decrease under drought and heat stress. Combined stress resulted in a much greater reduction (41.66%) in Fm/Fm. In addition, combined stress leads to a sharp decrease in quantum yield PSII (ΦPSII) and photochemical efficiency (qp), which is less than that under individual stresses. Additionally, under all stress conditions, an increase in non-photochemical (NPQ) quenching was observed.

Guidi and colleagues reported an Fv/Fm ratio in the range of 0.76 to 0.85 under normal conditions, which decreased under lowered conditions [41]. Reduced photosystem II activity (reduced PSII activity) has also been reported to lead to an imbalance between production and utilization of electrons in plants under saline conditions [42,43].

Salinity stress significantly reduced biomass accumulation, chlorophyll synthesis, photosynthesis, gas exchange parameters, and photochemical efficiency (Fv/Fm). Salinity stress leads to decreases in chlorophyll, photosynthetic rate (Pn), and Fv/Fm. Total chlorophyll, Pn, and Fv/Fm increased in plants under salinity stress in seedlings treated with NaCl + salicylic acid + nitric oxide [29].

### 2.2. Correlations between Chlorophyll Fluorescence Parameters

Under normal conditions, Fm had a positive and significant correlation with ET_o_/CS_o_, and TR_o_/CSm had a negative and significant correlation with ET_o_/RC.

Under drought conditions, Fm and Fv had a positive and significant correlation with ET_o_/CSm. Under saline conditions, Fm, Fo, and Fv were positively and significantly correlated with ET_o_/CSm and ET_o_/CS_o_.

Begum and colleagues showed a negative correlation between drought stress and fluorescence parameters, including PSII (Fv/Fm) efficiency, photochemical quenching (qP), and electron transport rate (ETR), whereas non-photochemical neutralization and non-photochemical quenching (NPQ) increased significantly compared to control plants [44].

All parameters related to chlorophyll fluorescence under salinity treatment were significantly reduced [45]. A negative correlation was reported between salinity stress and photosystem efficiency (PSII efficiency), Fv/Fm, and quantum yield (ΦPSII). In addition, under salinity-stress conditions, an increase in non-photochemical quenching (NPQ) has been observed [30,46,47].

### 2.3. Cluster Analysis of Barley Lines Based on Chlorophyll Fluorescence Parameters

**Figure 1:**
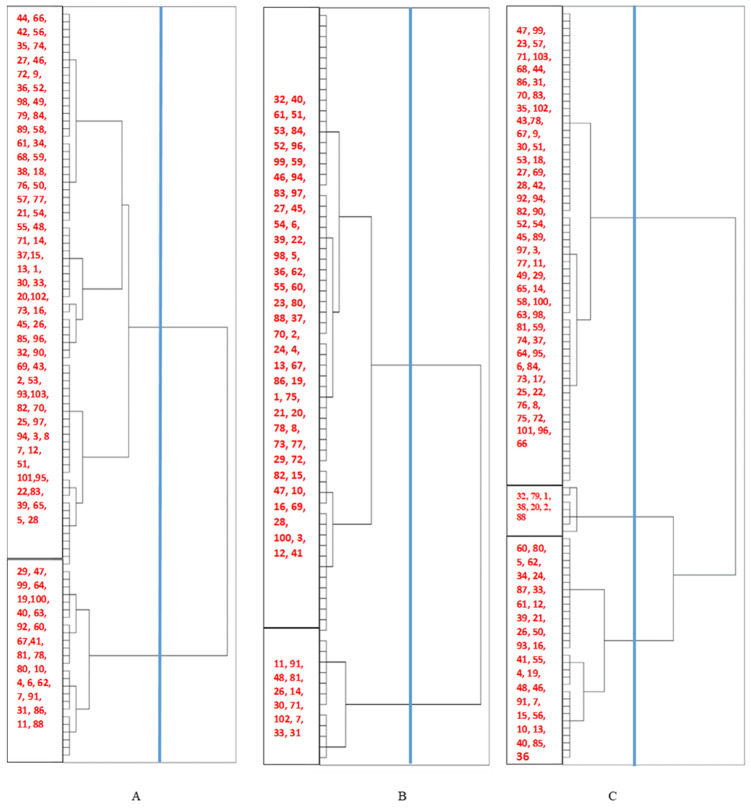
Clustering of barley recombinant inbred lines caused Badia × Kavir cross based on fluorescence chlorophyll attributes: (**A**) normal, (**B**) drought, and (**C**) salinity conditions.

Cluster analysis divided the studied lines under normal, drought, and saline conditions into two, two, and three groups, respectively (Figure 1, Table 3).

**Figure 2:**
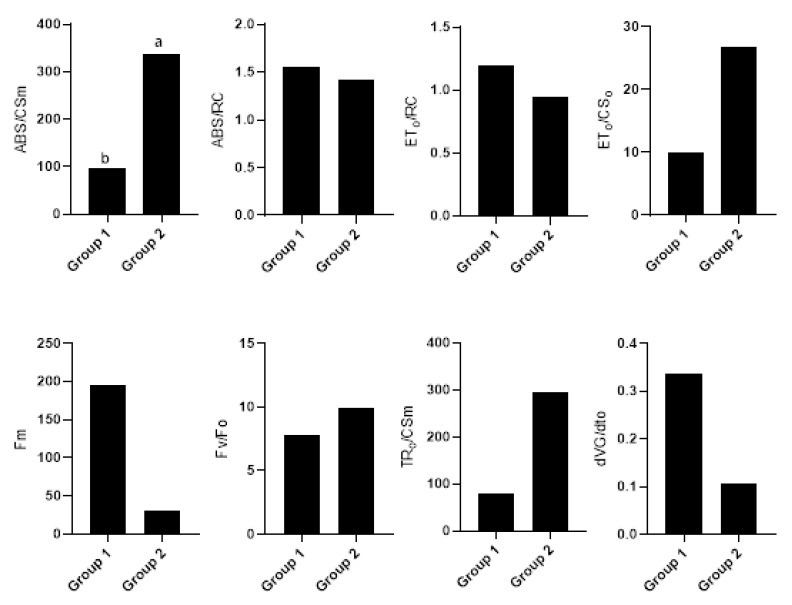
Mean comparison between groups created in the cluster analysis of normal conditions. Different letters indicate significant differences between groups.

**Figure 3:**
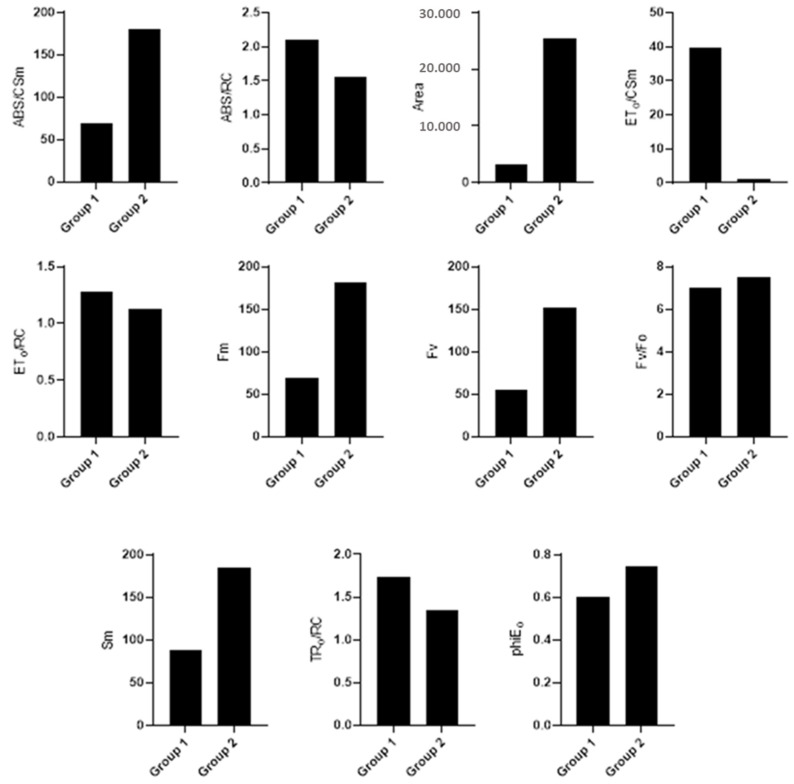
Mean comparison between groups created in the cluster analysis of drought conditions.

**Figure 4:**
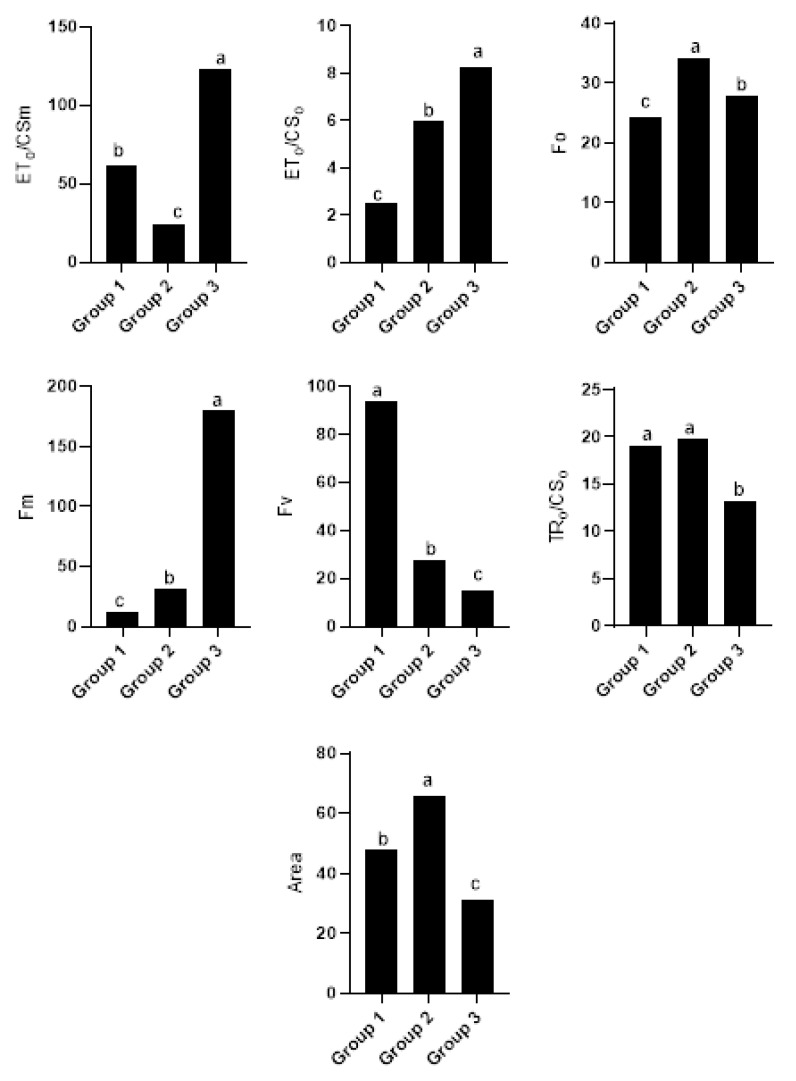
Mean comparison between groups created in the cluster analysis of salinity conditions. Different letters indicate significant differences between groups.

The differences between the groups created in the cluster analysis were significant for the chlorophyll fluorescence traits under normal conditions (Figure 2), drought (Figure 3), and salinity stresses (Figure 4). Accordingly, Pillai’s indicators trace, Wilks’ lambda, Hotelling’s trace, and Roy’s largest root were all significant at a 1% probability level (Table 3). Under normal conditions, the first group was more valuable in terms of ABS/RC, ET_o_/RC, and Fm than the second group and less valuable than the second group in terms of ABS/CSm, ET_o_/CS_o_, Fv/Fo, TR_o_/CSm, and dVGdt_o_. Once the lines were subjected to drought stress, the first group was more valuable than the second in terms of ABS/RC, ET_o_/CSm, ET_o_/RC, Fm, and TR_o_/RC. ABS/CSm, Area, Fv, Fv/Fo, Sm, and phiE_o_ were less valuable in terms of salinity stress in the second group.

### 2.4. Linkage Map Construction

**Figure 5:**
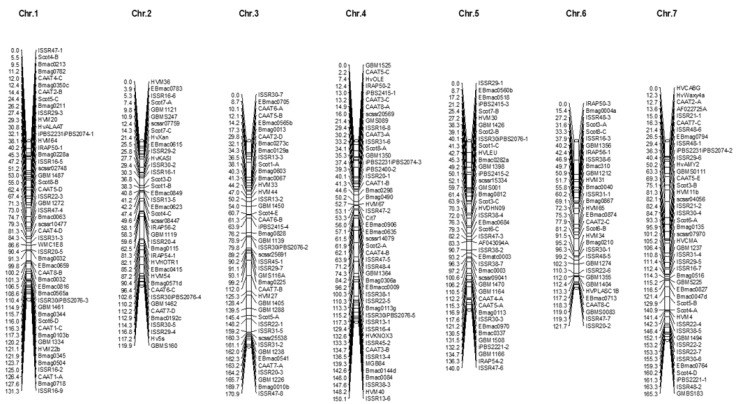
Linkage maps of barley chromosomes based on SSR, ISSR, IRAP, iPBS, CAAT, and Scott markers in RIL population based on Kavir and Badia Cross.

Linkage mapping was performed using 152 SSR markers, 72 ISSR alleles, seven IRAP alleles, 29 CAAT alleles, 27 Scot alleles, and 15 iPBS alleles (Figure 5). A Mendelian ratio (1:1) was examined for all the amplified alleles using the chi-squared test, and non-Mendelian transgressive segregation markers were not used for mapping. The molecular markers were assigned to seven linkage groups based on the seven barley chromosomes. Chromosome 1 covered 50 markers with a chromosome length of 131.3 cM. Additionally, 39, 44, 49, 42, 33, and 45 were assigned to chromosomes 2, 3, 4, 5, 6, and 7, with lengths of 190.9, 170.9, 150.1, 140.0, 121.7, and 165.3 cM, respectively (Figure 5). The arrangement of SSR markers was different from that previously reported [48,49,50,51], whereas their chromosome locations matched the maps.

The first linkage maps of barley were prepared by Graner et al. using 71 lines of double haploid populations from the Igri and Franka cross and RFLP markers [52]. Kleinhofs and collaborators prepared linkage maps using a double haploid population by crossing Steptoe and Morex and RFLP markers [53,54]. Wenzl and colleagues provided a linkage map using SSR, RFLP, STS, and DArT markers [53]. SSR, SST, and EST combined with AFLP have been used to prepare genetic linkage maps [51,55,56]. Finally, SNP markers, such as RFLP, SNP, and SSR, have been used by other authors for QTL mapping of barley populations [28,57,58].

### 2.5. Mapping Chlorophyll Fluorescence Attributes

#### 2.5.1. Mapping of Chlorophyll Fluorescence Attributes under Normal Conditions

A QTL (qABSRCN-7) was detected for ABS/RC on chromosome 7 at 104 cM between the scssr07970 and HVCMA, which explained 8.9% of the ABS/RC phenotypic variation. The qABSRCN-7 allele was transferred from Badia to the progenies and increased the ABS/RC by 0.749 (Table 4).

Furthermore, one QTL was detected on chromosome 5 between GBM1166 and IRAP54-2 at 136 cM for ET_o_/Cs_o_, which explained 10.6% of the ET_o_/Cs_o_ phenotypic variation. The qEToCSoN-5 allele of Kavir increased ET_o_/Cs_o_ by 5.688.

Only one QTL was detected for ET_o_/RC at 120 cM on chromosome 4 between ISSR13-1 and ISSR16-4, which explained 9.4% of ET_o_/RC phenotypic variation. The q EToRCN-4 allele was transferred from Kavir to the progeny, increasing the ET_o_/RC by 1.115, and one QTL (qFmN-5) on chromosome 5 at 136 cM for Fm was detected between GBM1166 and IRAP54-2. Increasing alleles of this QTL were transferred from Kavir to the progenies, affecting Fm (61.125).

One QTL was detected on chromosome 6 for Fv/Fo at 116 cM. This QTL (qFVFON-6) was located between the HVPLASC1B and EBmac0713 and explained 14.4% of the Fv/Fo phenotypic variation. The alleles of qFvFoN-6 were reduced from Badia to the progenies and reduced the value of Fv/Fo to −6.462. This QTL overlapped with the Fo-controlling QTL under stress conditions reported by Hassan et al. [59].

One QTL with an LOD of 2.076 was identified for TR_o_/CSm on chromosome 5 at 136 cM. qTR_o_CSmN-5 was located between GBM1166 and IRAP54-2, explaining 8.9% of TR_o_/CSm phenotypic variations. The qTR0CSmN-5 alleles were transferred from Badia to the progeny and had an effect of 52.319 on TR_o_/CSm. Two QTLs were detected on chromosome 2 for dVG/dt_o_ at 38 and 60 cM. The mentioned QTL explained 9.7% and 9.5% of the dVG/dt_o_ phenotypic variations, and the alleles of both were transferred from Kavir to the progenies; the values of dVG/dt_o_ were 1.376 and 0.967, respectively.

#### 2.5.2. Mapping of Chlorophyll Fluorescence Attributes under Drought Stress

Three QTLs (qABSRCD-4, qABSRCD-2, and qABSRCD-7) were detected in ABS/RC on chromosomes 4, 2, and 7, with LODs of 2.167, 2.262, and 2.401 at 48, 116, and 84 cM, respectively. These QTLs explained 12, 12.5, and 13.2% of ABS/RC phenotypic variations. Among the three QTLs tracked, one QTL was found to have an increasing effect (qABSRCD-4) of 2.573 and was transferred from Badia to the progenies. The other QTLs had a reducing effect of ABS/RC of −2.156 and −2.495 and were transferred from Kavir to progenies. In the studies of Guo et al. (2007), two genetic regions were responsible for Fo control under drought stress at the reproductive growth stage on chromosomes H2 and H7 at 116.15 and 85.2 cM, respectively [34].

The Area was controlled by five QTLs (LODs of 2.336, 2.384, 2.512, 2.139, and 3.017) on chromosomes 2, 3 (two QTLs), 6, and 7, at 6, 28, 128, 62, and 142 cM, respectively. Among them, one QTL (qAREAD-6) increased the area value by 4892.58 and was transferred from Badia to the progenies, explaining 9.1% of the phenotypic variation of Area. The other four QTLs, qAREAD-2, qAREAD-3a, qAREAD-3b, and qAREAD-7, demonstrated a decreasing effect of −8231.992, −5198.301, −3534.728, and −4127.683, respectively, which were transferred from Kavir to the progenies. These QTLs explained 9.9, 10.1, 10.6, and 12.6% of the phenotypic variations in the area. Only one QTL reduced the ET_o_/CSm ratio. This was detected between Bmag0013 and CAAT2-D on chromosome 3 at 28 cM, which explains 9.5% of ET_o_/CSm phenotypic variation. The reducing alleles of qEToCSmD-3 were transferred from Badia to the progenies and reduced to −28.284.

Two QTLs were detected for ET_o_/RC on chromosomes 1 and 7, with LODs 2.311 and 3.422 at 58 and 84 cM between Scot8-B and CAAT5-D, and also ISSR21-2 and ISSR30, which explained 12.8 and 18.3% of the phenotypic variation. qET_o_/RC-7 overlapped with the QTL controlling Fo, as previously reported [34]. The qET_o_RCD-1 allele was transferred from Kavir to the progenies and increased this parameter by 2.107, whereas the qET_o_RCD-7 allele decreased the value of ET_o_/RC by −2.007 and was transferred from Badia to the progenies.

For Fm, one QTL was detected on chromosome 3 at 38 cM between Bmag0013 and CAAT2-D, explaining 8.7% of the phenotypic variation in Fm. The qFmD-3 reducing allele was transferred from the parents to the progenies and reduced the Fm value by −43.24. Two QTLs were detected on chromosomes 3 and 6 at 28 and 94, respectively, in Fv. Moreover, 9.1% and 10% explained the phenotypic variation in Fv. qFvD-3 had a reducing role and was transferred from Badia to the progenies, reducing Fv by −36.668. Meanwhile, qFvD-6 had an increasing role and was transferred from Kavir to the progenies, increasing this parameter by 32.443. This QTL overlapped with the QTL controlling Fv under irrigated conditions [34].

A QTL (qFvFoD-2) was detected for Fv/Fo on chromosome 2 at 86 cM, located between EBmac0415 and HVM54, explaining 11% of the phenotypic variation in Fv/Fo. For Sm, one QTL was identified on chromosome 2 at 26 cM between ISSR29-2 and HvKASI, which explained 9.7% of the phenotypic variation in Sm. Increasing alleles qSmD-2 were transferred from Kavir to progenies and increased this parameter to 254.947. This QTL overlapped with the QTL controlling Fv/Fm under irrigated conditions, as detected by [33]. Two QTLs were detected for TR_o_/RC on chromosomes 2 and 7 at 48 and 84 cM between Scot4-C and scssr08447 and ISSR21-2 and ISSR30-4, respectively, which explained 12 and 13.6% of TR_o_/RC phenotypic variations. qET_o_RCD-2 and qET_o_RCD-7 reduced the TR_o_/RC by −1.523 and −1.792, respectively, and were transferred from Badia to progenies. [60]) reported that one QTL was responsible for controlling Fo and Fv/Fm on chromosome 2 at 44.3 cM under drought stress at the reproductive growth stage.

Two QTLs were detected on chromosomes 4 and 7 at 70 and 92 cM for PhiE_o_ between ISSR47-5 and ISSR48-4 and between ISSR30-4 and Scot6-A, respectively. These QTLs explained 11.7 and 9.4% of the PhiE_0_ phenotypic variation. qPHIE_o-_D4 and qPHIE_o-_D7 alleles were transferred to progenies from Badia, and PhiE_o_ values changed by −0.226 and −1.066, respectively.

#### 2.5.3. Mapping of Chlorophyll Fluorescence Attributes under Salinity Stress

Two QTLs were detected for the ABS/Cs_o_ and Fo on chromosomes 2 and 7 with LODs of 2.472 and 2.093 at 102 and 126 cM, respectively. It was located between CAAT6-C and ISSR30iPBS2076-4 and between Bmac0047 and Scot5-B, explaining 10.5 and 8.9% of the phenotypic variations in ABS/CS_o_ and Fo. The alleles belonging to qABSCS_o_S-2, qABSCS_o_S-7, qFoS-2, and qFoS-7 played increasing roles in ABS/Cs_o_ and Fo.

A QTL was detected for area (LOD = 2.141) on chromosome 7 at 126 cM. qAREAS-7 was located between Bmac0047 and Scot5-B and explained 9.1% of the phenotypic variation in the area. The qAREAS-7 enhancing allele was transferred from parental to progeny, increasing the area value by 12179.15. A QTL (LOD = 2.269) was detected for ET_o_/CSm between Bmac0047 and Scot5-B on chromosome 7 at 126 cM. This explains 9.6% of ET_o_/CSm phenotypic variation. Increasing alleles qET_o_CSmS-7 were transferred from Badia to the progenies, which increased ET_o_/CSm by 35.513. For ET_o_/CS_o_, one QTL (LOD = 2.295) was detected on chromosome 7 at 126 cM. qET_o_CS_o_S-7 was located between the Bmac0047- Scot5-B, which explained 9.8% of the phenotypic variation in ET_o_/CS_o_.

For Fm, two QTLs on chromosomes 2 and 7 were detected with LODs of 2.442 and 2.465 on chromosome 2, respectively, at positions 102 and 128 cM between CAAT6-C and ISSR30iPBS2076-4, and between Bmac0047 and Scot5-B, which explained 10.3% and 10.4% of the phenotypic variation. Increasing alleles qFmS-2 and qFmS-7 were transferred from Kavir to the progenies (86.526 and 59.741, respectively). In addition, two QTLs were detected for Fv on chromosomes 2 and 7 with LODs of 2.314 and 2.448, respectively, at 102 and 128 cM between CAAT6-C-ISSR30iPBS2076-4 and Bmac0047- Scot5 explaining 9.8% and 10.4% of the phenotypic variation. The alleles qFvS-2 and qFvS-7 had an increasing role and were transferred from Kavir to the progenies. qFvS-2 increased the amount of Fv to 70.813, and qFvS-7 increased it to 50.004. One QTL was detected on chromosome 2 at 102 cM for TR_o_/CS_o_. It was located between Bmac0047 and Scot5-B and explained 10.4% of TR_o_/CS_o_ phenotypic variation. Increasing alleles qTR_o_CS_o_S-2 were transferred from Kavir to the progenies.

The correlations among the traits might be attributed to the co-location of control QTLs or linkages between them. In addition, the genetic control of several traits with one gene or QTL is called pleiotropy. In this study, a similar phenomenon was observed. Under normal conditions, a region on chromosome 5 between GBM1166 and IRAP54-2 (at 136 cM), a common QTL, was detected for Fm, TR_o_/CSm, and ET_o_/CS_o_. In drought stress, the importance of the regions of chromosomes 3, 2, and 7 for controlling the traits associated with chlorophyll fluorescence was identified.

For area, ET_o_/CSm, Fm, and Fv, a common QTL was identified on chromosome 3 at 28 cM, flanked by Bmag0013 and CAAT2-D. In addition, one QTL was detected in the region of chromosome 2 for ABS/RC and TR_o_/RC at position (48) cM between Scot4-C and scssr08447.

Moreover, for ABS/RC, ET_o_/RC, and TR_o_/RC at position 84 cM on chromosome 7, a common QTL marker was detected between markers ISSR21-2 and ISSR30-4. The distance from this gene locus is slightly closer to 0.71 cM (ISSR30-4).

The regions on chromosomes 2 and 7 played a significant role in salinity stress. For ABS/CS_o_, Fm, Fo, Fv, and TR_o_/CS_o_ at 102 cM (cM) at chromosome 2, a QTL was detected between CAAT6-C and ISSR30iPBS2076-4. One QTL was located between Bmac0047-Scot5-B on chromosome 7 at 126 cM for ABS/CS_o_, Area, ET_o_/CSm, ET_o_/Cs_o_, and Fo. The closest marker was 3.87 (Scot5-B) cM.

For a long time, plant breeding relied on phenotypic features and was based on the selection of superior plants. When the genetic basis of the characteristic is reasonably straightforward, and the genetic influence of the gene is additive, selection based on the phenotype is successful. However, many important agronomic qualities are quantitative traits, such as the ability to tolerate abiotic stress or traits whose phenotypes are challenging to characterize precisely. Consequently, it is inaccurate to measure the genetic potential of a trait using the phenotype, and the selection process is ineffective. Based on the obtained results, we suggest the use of HVPLASC1B and EBmac0713 for normal conditions; Scot4-C—scssr08447, scssr08447, ISSR21-2, ISSR30-4, and HVM4 for drought stress; and Bmac0047, Scot5-B, and CAAT6-C in saline conditions for breeding programs, including genomic selection (GS), genome-wide selection (GWS), marker-assisted convergent crossing (MACC), marker-assisted gene pyramiding (MAGP), and marker-based backcrossing (MABC). The marker-assisted or marker-based backcross (MABC) is the simplest type of marker-assisted selection. Furthermore, one of the most significant uses of DNA markers for plant breeding is marker gene pyramiding (MAGP). By choosing two or more genes simultaneously, this method has been suggested and used to boost resistance to abiotic stresses. Marketer-assisted complex or convergent crossing (MACC) can be used to pyramid several genes and QTLs. The most crucial indicators for choosing lines with a high level of resistance to all abiotic stressors are introduced in this study.

## 3. Materials and Methods

### 3.1. Genetic Population

This study was conducted under greenhouse conditions at Gonbad Kavous University in 2019 using a completely randomized design with three replications. One hundred and six RIL barley lines caused Badia and Kavir crosses, were evaluated. Badia and Kavir were respectively susceptible and tolerant to abiotic stresses (salinity and drought) [61,62,63,64].

### 3.2. Seedling Growth

Mature seeds of uniform size were selected, and cultured pots (15 cm in diameter and 16 cm in height) were used; pots had a hole in the bottom for excessive water draining. The seeds were surface sterilized with 0.1% HgCl_2_ solution for 5 min and thoroughly washed with sterilized distilled water. Ten seeds were planted in each pot and covered with 1 cm of soil. All plants were grown in a growth chamber (WEISS TECHNIK, Germany, Balingen) with 16 h daylight at 28 °C, 8 h dark period at 20 °C, light intensity of 200 μmol m^−2^ s^−1^, light cycle of 12/12 h (light/dark), and around 70% relative humidity. Moreover, some of its physical and chemical properties were recorded through extraction with normal ammonium acetate (ratio of soil and ammonium acetate, 2:10), including soil texture extracted using the hydrometric method [65], pH (WTW 7110, Germany, Weilheim), and EC (Metrohm, Filderstadt, Germany) in saturated extract [60,66], and the percentage of carbon with organic carbon in the Walkely and Black method [67,68]. In this method, organic matter is oxidized by potassium dichromate (K_2_Cr_2_O_7_) with sulfuric acid (H_2_SO_4_) to heat the dilution (as the classical procedure) and potentiometric or colorimetric titration, equivalent to calcium carbonate with hydrochloric acid neutralization [69], and the total nitrogen in the soil was determined using the total nitrogen digestion method [70]. Total nitrogen was determined using the Kjeldahl digestion, distillation, and titration methods. Phosphorus was extractable with 0.5 M sodium bicarbonate using the Olsen method, and the absorption of potassium was recorded by extraction with normal ammonium acetate (Table 5). Water and seedling management were conducted regularly. When the seedlings were approximately 7 cm tall, each pot was thinned to only five seedlings of relatively consistent size with reasonable spacing.

### 3.3. Applying Salinity Stress

After 14 days of growth in non-saline soil, salinity stress was applied using saline water with a NaCl source at EC 8 dS m-1. Salt stress was raised to EC 16 dS m-1 after 7 d of growth at EC 8 dS m-1 to prevent plants from experiencing sudden shock. The salinity of the saturated extract was measured weekly in degraded pots. To prepare the saturated extract, approximately 150–100 g of potting soil was poured into a small plastic bucket. Distilled water was added. They were mixed with a spatula to form a paste or saturated paste. After preparing the saturated mud, we placed filter paper on a Buchner funnel, poured the saturated paste on it, and extracted the paste with a vacuum pump. A control set was maintained without salt addition.

### 3.4. Applying Drought Stress

The pots were filled with 2.2 kg of sterilized field soil, which contained approximately 6% water. The field capacity, wilting point, and available water content (AWC) of the soil were measured at the Gonbad Kavous University soil laboratory. For the experiment, 70 and 10% of AWC in the soil were considered as well-watered and severe drought conditions, respectively. AWC is the amount of water that a soil can store that is available for use by plants based on the potential rooting depth. It is the water held between the field capacity and the wilting point, adjusted downward for rock fragments and salts in solution [71,72]. To calculate the AWC, the soil was first placed on two ceramic plates with known porosity and was wetted to saturation. Then, ceramic plates were placed under two high-pressure chambers to extract water to the capacity of the farm (10 km) and up to the permanent wilting point (1500 kPa). After the sample was balanced at the target pressure, sample (c) was weighed, dried overnight in an oven at 105 °C, and weighed again after drying. Finally, the soil water content was calculated at each pressure, and the available water capacity was calculated as the soil water loss between 10 and 1500 kPa. The drought treatment was initiated after 14 days. The soil moisture for the pots under well-watered and drought-stress conditions was maintained with the required amount of water by weighing the pots and watering the plants every day. The days were counted after the AWC in the soil reached 10% to allow drought measurements at precisely determined intervals.

### 3.5. Chlorophyll Fluorescence Attributes

Fluorescence-activating light was provided with an array of three light-emitting diodes focused on the leaf surface to produce homogeneous light. All samples were adapted to darkness for 15 min prior to measurements. Generally, ChlF in vivo is measured after long (~20–30 min) dark adaptation. This usually allows QA, the primary stable electron acceptor of the photosystem (PS) II reaction center, to be fully oxidized and enables us to measure the minimal fluorescence (Fo). On the other hand, the maximal fluorescence (F_M_) is reached when all QA and all the electron carriers beyond it are in a reduced state. The kinetics of the rise from Fo to F_M_, the ChlF transient, are affected by the dynamics of the steps involved in PSII and PSI and beyond in photosynthesis. In general, when light is absorbed by dark-adapted plant leaves, PSII reaction centers close, and the ChlF yield rises from Fo to the peak, FP, during the first seconds of illumination, followed by a decline, ultimately leading to a steady-state fluorescence (FS) level in a few minutes [26].

Raw fluorescence OJIP transients were transferred to a spreadsheet using the HandyPEA program (PEA; Hansatech Instruments, King’s Lynn, UK). In this form, the data can be treated according to the equations of the JIP test parameters using any tabulation program [25,70]. The chlorophyll fluorescence parameters listed in Table 1 were recorded based on the explanations of Christen et al. [26].

### 3.6. Genotype Evaluations

After adding 400 microliter (µL) extraction buffer (50 mM HCl, pH 0.8, 2.5 mM EDTA, 300 mM NaCl, and 1% SDS), ground leaf samples were ready to be extracted. The contents were centrifuged for 10 min at 12,000× *g*/micro, and approximately 400 μL of the resulting lysate was mixed with 400 μL of chloroform. The supernatant above the water was transferred to another 1.5 mL tube, and the DNA was precipitated with ethanol. Afterward, the contents were centrifuged at full speed for 3 min, and the supernatants were discarded. The DNA strands were washed with 70% ethanol and then exposed to air, dried in 50 μL of TE buffer (10 mM Tris-HCl, pH 0.8, 1 mM EDTA, pH 0.8), and suspended.

The concentration of extracted DNA was estimated with a spectrophotometer, using OD_260/280_, and DNA quality and concentration were measured using 1.0% agarose gel electrophoresis, and adjustments were made for a final DNA concentration of 100 ng μL^−1^ and a total DNA over 50 μg.

Three hundred and sixty-three SSR markers appropriately distributed over seven chromosomes in barley were selected according to previous studies [48,49,50,51,57,73]. These SSR primer pairs were examined for polymorphisms between the two parents, and polymorphic primers were used to amplify the DNA of each plant from the RIL population. One hundred and fifty-two SSR polymorphic markers were used to prepare a primary map. Polymerase chain reaction (PCR) was applied for SSR markers using 50 ng DNA, 0.67 M primers, 10 M reaction buffer, 2.5 M MgCl, 0.2 m dNTP, 0.5 units of Taq DNA polymerase, and deionized water in a volume of 15 μL. DNA amplification was performed according to the following program: denaturation at 94 °C for 5 min, followed by 30 cycles of denaturation at 94 °C for 1 min, annealing at 58 °C for 1 min, elongation at 72 °C for 1.5 min, and final extension at 72 °C for 5 min. Polymerase chain reaction (PCR) was performed using a thermocycler (iCycler BIORAD, USA). The amplified product was isolated on polyacrylamide gel electrophoresis 6% (PAGE) and made visible by silver staining. To saturate the markers’ primary maps, we employed iPBS [74], IRAP [75,76], ISSR (University of British Columbia, UBC), ScoT [77], and CAAT [78]. To investigate the polymorphism of the above-mentioned markers between parents, 21 IPBS markers, 8 IRAP markers, 15 SCoT markers, 15 CAAT markers, and 1 combination of ISSRiPBS markers were used. Ultimately, 7 IRAP alleles, 29 CAAT alleles, 27 SCoT alleles, 72 ISSR alleles, 15 IPBS alleles, and 5 combinations of ISSRiPBS polymorphic alleles were used for linkage map construction. For genetic mapping, we applied scores of 1 (for the male parent band) and 2 (for the female parent band) for SSR markers. For ISSR, iPBS, IRAP, SCoT, and CAAT markers, we used scores of 1 (for the presence of the band) and 3 (for the absence of the band) once the band was observed in the paternal parent and scores of 2 (for the presence of the band) and 4 (for the absence of the band) when the band was present in the parent.

### 3.7. Statistical Analysis, Linkage Map Construction, and QTL Analysis

#### 3.7.1. Analysis of Phenotypic Data

Pearson’s correlation between chlorophyll fluorescence parameters and cluster analysis of barley lines was performed based on the Euclidean distance squared and Ward’s method. Statistical analyses were performed using the SPSS 24 software.

#### 3.7.2. Analysis of Molecular Data

IPBS, IRAP, ISSR, SCoT, CAAT, and polymorph SSR markers were evaluated using the QGene [79] program individually with the χ2 test for a 1:1 segregation ratio for the RIL population [80] at a probability level of 0.01. Map preparation was carried out using the QTX17 software. All marker positions were in good agreement with those in previous maps [48,49,50,51,57,81]. Map distances (cM) based on the Kosambi function and a critical LOD threshold of 2 were used to determine the linkage groups. QGENE 4 software [79] was used to identify the QTL. The composite interval mapping (CIM) method was used to determine QTLs and estimate their effects, and the point with the highest LOD was identified as the region with the highest probability of QTL.

## 4. Conclusions

In this study, we showed that the gene regions detected for chlorophyll fluorescence parameters in barley were specific under normal, drought, and salinity conditions. We proved that if the plant is subjected to drought stress, certain gene regions will be expressed in its tolerance to stress, and other gene regions will be activated in salt-stress conditions. Seven regions consisting of QTLs were mapped onto linkage groups 1H to 7H, which were active for more than one attribute. QTLs for chlorophyll fluorescence were clustered into different groups in specific genomic regions. We also discovered several gene regions where genes controlling several traits were located in the same region. This overlapping can lead us to use the gene pyramiding technique. Gene pyramiding is an important strategy for crop improvement. The pyramid requires breeders to consider the minimum population size that must be assessed to have a reasonable chance of achieving the desired resulting genotype. Genotyping of molecular markers can facilitate the process of pyramiding genes by reducing the number of generations that breeders must evaluate to ensure that they have the desired gene combination. Also, this QTL-locating project for chlorophyll fluorescence provides valuable information on genomic regions that may be useful for marker-assisted breeding for drought and salinity tolerance improvement at the seedling stage in barley. Finally, further studies are needed to validate the QTLs detected in this study in more diverse genetic populations and more molecular markers so that the results of this study can be used to improve barley tolerance to drought and salinity stresses.

## Figures and Tables

**Table 1:** The list of the parameters for chlorophyll fluorescence [26].

Fo	Minimum fluorescence
Fv	Variable fluorescence
FM	Maximum fluorescence
FV/FM	Maximum quantum yield (photochemical efficiency) of primary PSII photochemistry; photochemistry in the dark-adapted state
Fv/Fo	The ratio of maximum quantum yield of photochemistry (FV/Fm) and competitive non-photochemical processes; (Fo/Fm) of PSII in the dark-adapted state; lateral reactivity of PSII
Area	Total complementary area between fluorescence induction (OJIP) curve and the line F = FM
Sm	Normalized total complementary area above the OJIP transient (reflecting multiple turnover QA reduction events) or total electron carriers per RC; Sm = area/(Fm–Fo)
**Phenomenological fluxes or activities per excited cross-section**	
ABS/CS_o_	Absorption flux per cross-section (CS) at time zero (t = 0); ABS/CS0 = Fo
ET_o_/CS_o_	Electron transport flux per CS at time zero (t = 0); ET_o_/CS_o_ = [1 − (Fo/Fm)] (1 − Vj) Fo
TR_o_/CS_o_	Trapped energy flux per CS at time zero (t = 0); TR_o_/CS_o_ = (TR0/ABS) (ABS/CS0) = Fo [1 − (Fo/Fm)]
DI_o_/CS_o_	Dissipated energy flux per CS at time zero (t = 0); DI_o_/CS_o_ = (ABS/CS0) − (TR_o_/CS_o_) = Fo − {Fo [1 − (Fo/Fm)]}
ABS/CSm	Light energy absorption flux per excited CS; approximated by FM; ABS/CSm = Fm
DI_o_/CSm	Dissipated energy flux (non-photochemical quenching) per excited cross-section; DIo/CSm = (ABS/CSm) - (TR_o_/CSm) = Fm - {Fm [1 - (Fo/Fm)]}; approximated by FM
TR_o_/CSm	Trapped energy flux per excited cross-section; TR_o_/CSm = (TR_o_/ABS) (ABS/CSm) = Fm [1 − (Fo/Fm)]; approximated by FM
ET_o_/CSm	Electron transport flux per CS
	Election transport flux per excited cross-section; the amount of energy used for the electron transport; ET_o_/CSm = [1 − (Fo/Fm)] (1 − Vj) Fm; approximated by FM
RE_o_/CSm	The flux of electrons from QA to final PSI acceptors per cross-section of PSII at maximum time; RE_o_/CS_o_ = [1 - (Fo/Fm)] (1 - Vj) [(1 - Vi)/(1 - Vj)] Fm
**Specific fluxes or activities per reaction center**	
ABS/RC	Absorption flux (for PSII antenna chls) per reaction center (RC); light energy absorbed by RC; ABS/RC = Mo·(1/VJ)·[1/(Fv/Fm)]
ET_o_/RC	Electron transport flux per active reaction center at t = 0; ET_o_/RC = Mo·(1/VJ)·(1–VJ)
DI_o_/RC	Dissipation energy flux per active reaction center at t = 0; DI_o_/RC = (ABS/RC)–(TR_o_/RC)
TR_o_/RC	Trapped energy flux per reaction center (at t = 0); TR_o_/RC = Mo·(1/VJ)
RE_o_/RC	Electron flux leading to the reduction in the PSI end acceptor per reaction center; REo/RC = Mo (1/Vj) (1 − Vi)
psi(Eo)	Quantum function of electron current
phi(Po)	Primary photochemical quantum performance
vj	Relative variable fluorescence at phase J of the fluorescence induction curve (2 ms); VJ = (FJ–Fo)/(Fm–Fo)
vi	Relative variable fluorescence at phase I of the fluorescence induction curve (30 ms); VI = (FI–Fo)/(Fm–Fo)
Dv/dt_o_	Initial slope of relative variable fluorescence, which expresses the rate of the RCs’ closure
DVG/dt_o_	Expresses the excitation

**Table 2:** Comparison of means for chlorophyll fluorescence parameters under normal conditions and drought and salinity stresses. In parentheses is the amount of increase and decrease in the attributes due to stress.

	Normal	Drought			Salinity			F	Sig.
	RILs	Badia	Kavir	RILs	Badia	Kavir	RILs		
Area	23,923.2 ^c^	2634.25	21,824.1	25,698.3 ^b^ (+7.42%)	27,951.3	25,614.2	26,874.3 ^a^ (+12.34%)	5.144	0.007
Fo	21.835 ^c^	22.624	17.325	28.632 ^a^ (+31.13%)	27.087	23.901	26.049 ^b^ (+19.30%)	4.832	0.009
Fm	151.233 ^a^	138.121	169.218	69.155 ^c^ (−54.28%)	68.371	75.078	72.365 ^b^ (−52.15%)	6.553	0.002
Fv	129.398 ^a^	121.367	136.847	56.845 ^c^ (−56.07%)	57.137	61.081	58.396 ^b^ (−54.87%)	6.785	0.001
Fv/Fm	0.822 ^a^	0.678	1.024	0.785 ^b^ (−4.50%)	0.617	0.794	0.712 ^c^ (−13.38%)	8.605	0.000
Fv/Fo	8.829 ^a^	8.245	9.857	4.632 ^c^ (−47.54%)	4.379	67.037	5.506 ^b^ (−62.36%)	2.704	0.069
Vj	0.154 ^b^	0.124	0.196	0.204 ^a^ (+32.47%)	0.201	0.254	0.208 ^a^ (+35.04%)	2.291	0.014
Vi	0.372 ^a^	0.256	0.581	0.265 ^b^ (−28.76%)	0.238	0.319	0.274 ^b^ (−26.34%)	4.444	0.013
dVdto	0.184 ^c^	0.142	0.196	0.326 ^a^ (+77.17%)	0.208	0.319	0.253 ^b^ (+37.5%)	5.847	0.003
Sm	162.287 ^a^	153.25	164.078	87.627 ^c^(−46.00%)	95.429	63.153	98.365 ^b^ (−39.39%)	2.496	0.085
ABS/RC	1.545 ^c^	1.597	1.325	2.067 ^b^ (+33.79%)	3.674	2.927	3.017 ^a^ (+95.27%)	1.524	0.015
DIo/RC	0.207 ^c^	0.235	0.168	0.304 ^b^ (+46.86%)	0.519	0.345	0.405 ^a^ (+95.65%)	1.068	0.018
TRo/RC	1.337 ^a^	1.279	1.428	1.199 ^a^ (−10.32%)	1.145	1.627	1.298 ^a^ (−2.917%)	1.591	0.206
ETo/RC	1.147 ^a^	1.134	1.217	1.009 ^a^ (−12.03%)	0.964	1.627	1.043 ^a^ (−9.07%)	0.500	0.307
ABS/CSo	21.835 ^b^	22.373	17.965	27.395 ^a^ (+25.46%)	28.090	23.298	26.049 ^a^ (+19.30%)	4.832	0.009
DIo/CSo	3.927 ^c^	4.974	3.457	5.961 ^a^ (+51.79%)	6.974	3.607	4.965 ^b^ (+26.43%)	3.153	0.045
TRo/CSo	17.908 ^a^	15.324	18.473	9.568 ^c^ (−46.57%)	10.258	13.817	12.685 ^b^ (−29.16%)	5.297	0.006
ETo/CSo	13.690 ^a^	11.142	14.374	6.986 ^c^ (−48.97%)	8.634	9.724	8.965 ^b^ (−35.51%)	5.217	0.006
ABS/CSm	151.233 ^a^	143.95	153.278	69.155 ^c^ (−54.27%)	71.259	78.089	75.962 ^b^ (−49.77%)	6.553	0.002
DIo/CSm	21.835 ^b^	23.143	20.765	28.964 ^b^ (+32.65%)	29.033	21.374	26.049 ^b^ (+19.30%)	4.832	0.109
TRo/CSm	129.398 ^a^	125.124	131.089	56.845 ^b^ (−56.07%)	54.234	62.300	59.362 ^b^ (−54.12%)	6.785	0.001
ETo/CSm	105.631 ^a^	104.087	109.143	98.362 ^b^ (−6.88%)	92.674	96.667	94.049 ^c^ (−10.96%)	6.804	0.001
REo/CSm	74.709 ^a^	70.329	77.283	30.214 ^c^ (−59.56%)	51.089	56.973	56.262 ^b^ (−24.69%)	6.151	0.003

Different letters in each row indicate differences between RILs means under normal, drought and salinity conditions. The full forms of the trait abbreviations are presented in Table 1.

**Table 3:** Multivariate analysis of variance in normal, drought, and salinity conditions.

Statistics	Statistics Value	F	*p*-Value
Normal			
Pillai’s Trace	0.768	42.671	≤0.0001
Wilks’ Lambda	0.232	42.671	≤0.0001
Hotelling’s Trace	3.319	42.671	≤0.0001
Roy’s Largest Root	3.319	42.671	≤0.0001
Drought			
Pillai’s Trace	0.855	33.619	≤0.0001
Wilks’ Lambda	0.145	33.619	≤0.0001
Hotelling’s Trace	5.898	33.619	≤0.0001
Roy’s Largest Root	5.898	33.619	≤0.0001
Salinity			
Pillai’s Trace	0.872	12.367	≤0.0001
Wilks’ Lambda	0.184	21.106	≤0.0001
Hotelling’s Trace	4.14	32.432	≤0.0001
Roy’s Largest Root	4.066	65.052	≤0.0001

**Table 4:** Detected putative QTLs for chlorophyll fluorescence attributes in the F8 population derived from Badia × Kavir cross by composite interval mapping methods.

Traits	QTL ^a^	Chr	Position	Flanking Markers	Distance to Closer Marker	LOD	Additive Effect	R^2^	Allele Direction
				Normal					
ABS/RC	q ABSRCN-7	7	104	scssr07970—HVCMA	1.21(HVCMA)	2.012	0.749	8.9	BADIA
ET_o_/CS_o_	qET_o_CS_o_N-5	5	136	GBM1166—IRAP54-2	0.27(IRAP54-2)	2.515	5.688	10.6	KAVIR
ET_o_/RC	q ET_o_RCN-4	4	120	ISSR13-1—ISSR16-4	2.74(ISSR13-1)	2.186	1.115	9.7	KAVIR
Fm	qFMN-5	5	136	GBM1166—IRAP54-2	0.27(IRAP54-2)	2.216	61.125	9.4	KAVIR
Fv/Fo	qFVF_o_N-6	6	116	HVPLASC1B—EBmac0713	1.23(EBmac0713)	3.443	−6.462	14.4	BADIA
TR_o_/CSm	qTR_o_CSmN-5	5	136	GBM1166—IRAP54-2	0.27(IRAP54-2)	2.076	52.317	8.9	KAVIR
dVG/dt_o_	qdVGdt_o_N-2a	2	38	Scot3-D—Scot1-B	0.25(Scot1-B)	2.27	1.376	9.7	KAVIR
	qdVGdt_o_N-2b	2	60	ISSR20-4—Bmag0115	0.39(ISSR20-4)	2.228	0.967	9.5	KAVIR
				Drought					
ABS/RC	q ABSRCD-2	2	48	Scot4-C—scssr08447	0.64(Scot4-C)	2.167	−2.156	12	KAVIR
	q ABSRCD-4	4	116	ISSR30iPBS2076-5—ISSR13-1	0.77(ISSR30iPBS2076-5)	2.262	2.573	12.5	BADIA
	q ABSRCD-7	7	84	ISSR21-2—ISSR30-4	0.71(ISSR30-4)	2.401	−2.495	13.2	KAVIR
Area	qAREAD-2	2	6	ISSR16-6—Scot7-A	0.75(ISSR16-6)	2.336	−8231.992	9.9	KAVIR
	qAREAD-3a	3	28	Bmag0013-CAAT2-D	1.78(CAAT2-D)	2.384	−5198.301	10.1	KAVIR
	qAREAD-3b	3	128	HVM27-GBM1405	0.41(GBM1405)	2.512	−3534.728	10.6	KAVIR
	qAREAD-6	6	62	ISSR31-1—Bmag0867	1.77(ISSR31-1)	2.139	4892.58	9.1	BADIA
	qAREAD-7	7	142	HVM4—ISSR22-4	0.32(ISSR22-4)	3.017	−4127.683	12.6	KAVIR
ET_o_/CSm	q ET_o_CSmD-3	3	28	Bmag0013-CAAT2-D	1.78(CAAT2-D)	2.23	−28.844	9.5	BADIA
ET_o_/RC	qET_o_RCD-1	1	58	Scot8-B—CAAT5-D	3.06(Scot8-B)	2.311	2.107	12.8	KAVIR
	qET_o_RCD-7	7	84	ISSR21-2—ISSR30-4	0.71(ISSR30-4)	3.422	−2.007	18.3	BADIA
Fm	qFmD-3	3	28	Bmag0013-CAAT2-D	1.78(CAAT2-D)	2.03	−43.24	8.7	BADIA
Fv	qFVD-3	3	28	Bmag0013-CAAT2-D	1.78(CAAT2-D)	2.127	−36.668	9.1	BADIA
	qFVD-6	6	94	HVM34—Bmag0210	1.23(Bmag0210)	2.364	32.434	10	KAVIR
Fv/Fo	q FvFoD-2	2	86	EBmac0415—HVM54	0.79(EBmac0415)	2.148	−9.559	11	BADIA
Sm	q SmD-2	2	26	ISSR29-2—HvKASI	0.24(ISSR29-2)	2.203	254.947	9.7	KAVIR
TR_o_/RC	q ET_o_RCD-2	2	48	Scot4-C—scssr08447	0.64(Scot4-C)	2.162	−1.523	12	BADIA
	q ET_o_RCD-7	7	84	ISSR21-2—ISSR30-4	0.71(ISSR30-4)	2.482	−1.792	13.6	BADIA
phiEo	qPHIEoD-4	4	70	ISSR47-5—ISSR48-4	1.15(ISSR48-4)	2.784	−0.226	11.7	BADIA
	qPHIEoD-7	7	92	ISSR30-4—Scot6-A	1.25(Scot6-A)	2.205	−1.066	9.4	BADIA
				Salinity					
ABS/CS_o_	qABSCS_o_S-2	2	102	CAAT6-C—ISSR30iPBS2076-4	0.58(ISSR30iPBS2076-4)	2.472	15.713	10.5	BADIA
	qABSCS_o_S-7	7	126	Bmac0047—Scot5-B	3.87(Scot5-B)	2.093	9.707	8.9	BADIA
Area	qAREAS-7	7	126	Bmac0047—Scot5-B	3.87(Scot5-B)	2.141	12,179.15	9.1	BADIA
ET_o_/CSm	qET_o_CSmS-7	7	126	Bmac0047—Scot5-B	3.87(Scot5-B)	2.269	35.513	9.6	KAVIR
ET_o_/CS_o_	qET_o_CS_o_S-7	7	126	Bmac0047—Scot5-B	3.87(Scot5-B)	2.295	5.291	9.8	KAVIR
FM	qFMS-2	2	102	CAAT6-C—ISSR30iPBS2076-4	0.58(ISSR30iPBS2076-4)	2.442	86.526	10.3	KAVIR
	qFMS-7	7	128	Bmac0047—Scot5-B	1.87(Scot5-B)	2.465	59.741	10.4	KAVIR
Fo	qFoS-2	2	102	CAAT6-C—ISSR30iPBS2076-4	0.58(ISSR30iPBS2076-4)	2.472	15.713	10.5	BADIA
	qFoS-7	7	126	Bmac0047—Scot5-B	1.87(Scot5-B)	2.093	9.707	8.9	BADIA
FV	qFVS-2	2	102	CAAT6-C—ISSR30iPBS2076-4	0.58(ISSR30iPBS2076-4)	2.314	70.813	9.8	KAVIR
	qFVS-7	7	128	Bmac0047—Scot5-B	1.87(Scot5-B)	2.448	50.004	10.4	KAVIR
TR_o_/CS_o_	qTR_o_CS_o_S-2	2	102	CAAT6-C—ISSR30iPBS2076-4	0.58(ISSR30iPBS2076-4)	2.45	12.58	10.4	KAVIR

^a^: Quantitative Traits Loci.

**Table 5:** Soil physical and chemical properties at the experiment site (0–30 cm depth).

Sand (%)	Silt (%)	Clay (%)	Potassium (ppm)	Phosphorus (ppm)	N (%)	Organic Carbon (%)	Neutral Substances (%)	pH	EC (dS/m)
13	58	29	316	11.4	0.09	0.90	9.5	7.6	1.19

## Data Availability

The data will be made available on reasonable request.
